# Patient Admission Preferences and Perceptions

**DOI:** 10.5811/westjem.2015.7.27458

**Published:** 2015-10-20

**Authors:** Clayton Wu, Joy Melnikow, Tu Dinh, James F. Holmes, Samuel D. Gaona, Thomas Bottyan, Debora Paterniti, Daniel K. Nishijima

**Affiliations:** * University of California, Davis, School of Medicine, Department of Emergency Medicine, Sacramento, California; † University of California, Davis, School of Medicine, Center for Health Care Policy and Research, Sacramento, California

## Abstract

**Introduction:**

Understanding patient perceptions and preferences of hospital care is important to improve patients’ hospitalization experiences and satisfaction. The objective of this study was to investigate patient preferences and perceptions of hospital care, specifically differences between intensive care unit (ICU) and hospital floor admissions.

**Methods:**

This was a cross-sectional survey of emergency department (ED) patients who were presented with a hypothetical scenario of a patient with mild traumatic brain injury (TBI). We surveyed their preferences and perceptions of hospital care related to this scenario. A closed-ended questionnaire provided quantitative data on patient preferences and perceptions of hospital care and an open-ended questionnaire evaluated factors that may not have been captured with the closed-ended questionnaire.

**Results:**

Out of 302 study patients, the ability for family and friends to visit (83%), nurse availability (80%), and physician availability (79%) were the factors most commonly rated “very important,” while the cost of hospitalization (62%) and length of hospitalization (59%) were the factors least commonly rated “very important.” When asked to choose between the ICU and the floor if they were the patient in the scenario, 33 patients (10.9%) choose the ICU, 133 chose the floor (44.0%), and 136 (45.0%) had no preference.

**Conclusion:**

Based on a hypothetical scenario of mild TBI, the majority of patients preferred admission to the floor or had no preference compared to admission to the ICU. Humanistic factors such as the availability of doctors and nurses and the ability to interact with family appear to have a greater priority than systematic factors of hospitalization, such as length and cost of hospitalization or length of time in the ED waiting for an in-patient bed.

## INTRODUCTION

The Institute of Medicine emphasizes that “desired outcomes” are a composite of patient and clinical goals so that care is patient-centered -- respectful of and responsive to individual patient preferences, needs, and values. 1 Quality measurements and improvement efforts often focus on clinical processes and outcomes of care, such as hospital complications, time to intervention, and risk-adjusted mortality. These measures, however, do not capture other outcomes that are important to patients and their caregivers.

Patients’ experiences during hospitalization are an important aspect of delivering quality care. The Centers of Medicare and Medicaid Services have prioritized this aspect of care by including measurements of patient hospitalization experiences as part of hospital reimbursement.^2^

A significant factor impacting patients’ experiences during hospitalization is the type of hospital unit (most commonly the intensive care unit [ICU], a telemetry floor, or a general floor) to which they are admitted. Numerous differences exist between the ICU and the hospital floors, all of which may impact patients’ experiences during hospitalization. For example, a higher frequency of vital sign measurements in the ICU compared to the floor may facilitate more frequent data on patient status but may also impact a patient’s privacy and ability to sleep.

For patients who clearly benefit from ICU care (i.e., those who are severely ill and/or unstable), admission to the floor is not a viable option as the clinical outcome benefits strongly favor the ICU. 3–6 However, many patients are admitted to the ICU primarily for observation and are at low risk for requiring a critical care intervention such as mechanical ventilation or vasopressor infusion. 7 For these patients, the clinical outcome benefits of ICU admission are much less evident, and other patient-centered outcomes such as their experiences during hospitalization should be considered.

Understanding patient perceptions and preferences of hospital care is important to improve patients’ hospitalization experiences and satisfaction. The objective of this study was to conduct a patient survey based on a hypothetical scenario of mild traumatic brain injury (TBI) to investigate patient preferences and perceptions of hospital care, specifically differences between ICU and hospital floor admissions.

## METHODS

### Study Design

This was a cross-sectional survey of emergency department (ED) patients conducted at a Level I trauma center. Patients were presented a hypothetical scenario of a patient with mild TBI and were surveyed about preferences and perceptions of hospital care related to this scenario. This study was approved by the study site’s institutional review board. The study was anonymous (no patient identifiers were collected) and all patients gave verbal consent to participate in the study.

### Study Setting and Population

The study population consisted of a convenience sample of ED patients surveyed between December 2012 and March 2013. Adult (18 years and older) ED patients in the ED waiting room who spoke English as their primary language were eligible. Excluded patients included those presenting to the ED for psychiatric evaluation, prisoners or those who were in custody, intoxicated patients, patients with a history of dementia or altered level of consciousness, and pregnant patients. Patients were enrolled seven days a week from 5 a.m. to midnight.

### Survey Development

We developed two separate questionnaires for the study: one consisted of closed-ended questions to provide quantitative data on patient preferences and perceptions of hospital care, and the second consisted of three open-ended questions to evaluate factors or themes that may not have been captured with the closed-ended questionnaire (see Appendix). The questionnaires used the general framework of the Hospital Consumer Assessment of Healthcare Providers and Systems (HCAHPS) survey, which measures hospitalized patients’ perspectives on different hospital experience topics such as nurse and doctor communication, responsiveness of hospital staff, and quietness of hospital environment.^2^ The HCAHPS survey is the first national, standardized, publicly reported survey of patients’ perspectives of hospital care and was developed through a rigorous and multi-faceted scientific process including a public call for measures, literature review, cognitive interviews, consumer focus groups, stakeholder input, a three-state pilot test, extensive psychometric analyses, consumer testing, and numerous small-scale field tests.

Both questionnaires were drafted by two of the study authors (CW and DN) and revised based on input from all of the study authors. The questionnaires were then administered to 10 patients who provided feedback on question style, wording, and content (cognitive testing) and 10 separate patients who provided feedback on the logistical aspects of administering the questionnaires, including screening, determination of inclusion and exclusion criteria, question order, and the overall length of the questionnaires (pilot testing). We refined the questionnaires after each stage of testing.

### Closed-ended Questionnaire Protocol

The closed-ended questionnaire was administered to 302 eligible patients. We considered this sample size adequate to generate sufficiently narrow confidence intervals (CIs). The questionnaire included background questions, a clinical scenario, and multiple choice questions. Background questions evaluated relevant patient characteristics including self-reported general health, race, ethnicity, education level, insurance status, and prior experiences with ED and in-hospital care. Patients then read a clinical scenario where they suffered a TBI with a small intracranial hemorrhage diagnosed on head computed tomography (Figure). This particular clinical scenario involving TBI was chosen because we previously demonstrated that many low-risk patients with TBI and intracranial hemorrhage likely do not require ICU admission and wide variability of ICU admission practices exists across trauma centers. 8–11

Multiple choice questions addressed patient preferences and perceptions of hospital care in the context of the clinical scenario. Patient preference questions (10 questions) evaluated the importance (five-point scale; very important to not important at all) of specific hospitalization factors including access to family and friends, access to treating doctor or nurse, cost, ED and hospital length of stay, privacy, and ability to sleep. Patients were also asked choose the most important hospitalization factor and their preference of admission location (ICU, floor, or no preference). Perception-of-care questions (14 questions) addressed perceived differences between ICU and floor admission along the same hospitalization factors. Questions and answer choices were read aloud to the patient by trained research associates while the patient marked answers on a paper questionnaire.

### Open-ended Questionnaire Protocol

The open-ended questionnaire was administered until theme saturation was reached (30 patients). Sampling was conducted the same way as with the closed-ended questionnaire and consisted of the same patient background questions and clinical scenario provided in the closed-ended questionnaire. Patients were not asked the closed-ended questions because they may have influenced responses to the open-ended questions. The three open-ended questions were:

- “If you were the patient in the scenario, would you prefer to be in the ICU or the floor? Why?”
- “How do you think hospitalization in the ICU versus on the floor differ?”
- “If you had to be admitted to the hospital, what factors are important to you?”

Survey questions were read aloud to the patient and their verbal responses were audio-recorded and transcribed.

### Analysis

We conducted data formatting and recoding of variables using STATA 11.0 statistical software (STATA Corp, College Station, TX). The study population was described using descriptive statistics. We reported normal data with means and standard deviations and proportions were presented with 95% CIs.

For the open-ended questionnaire, transcriptions were uploaded into ATLAS.ti (Belin, Germany), a qualitative data analysis software program. Transcripts were reviewed by two authors (DN and JM) who independently generated an exhaustive list of items representing emergent themes and factors regarding patient preferences, perceived differences between the ICU and the floor, and hospitalization factors important to patients. This exhaustive list was narrowed to generate a summative list of themes and factors. We developed coding criteria and systematically applied them to the formatted transcripts by “tagging” elements within the transcripts. “Tagged” elements were quantitatively assessed to identify predominant factors and common themes. We then chose from the transcripts specific quotes that best represented these factors and themes.

## RESULTS

### Characteristics of Study Subjects

A total of 332 patients were enrolled in the study; 302 patients completed the closed-ended questionnaire and 30 patients completed the open-ended questionnaire. There were 143 males (44%) and the mean age was 44.3 years (SD 14.9 years). Two-hundred seventy three of 317 patients (86%) responded that they had some form of insurance and 210 of 317 (66%) patients said they were previously admitted to a hospital. See Table 1 for complete patient characteristics.

### Main Results

#### Importance of Hospitalization Factors

On the closed-ended questionnaire, the ability for family and friends to visit (83%), nurse availability (80%), and physician availability (79%) were the factors with the highest response of “very important,” while the cost of hospitalization (62%) and length of hospitalization (59%) received the lowest response (Table 2 and eTable 1). When asked to choose which of the eight factors is the most important during hospitalization, 54% choose physician availability followed by the ability for family and friends to visit (14%) (Table 2).

The open-ended questionnaire revealed six summative categories of important hospitalization factors. This list outlines these categories with representative patient quotations.

1. Availability to family and friends
  “One of the most important things to me would be being able to visit my family.”
  “My wife can seye me when she wants to.”
2. Competency of doctors and nurses
  “Quality of the physicians, nurses, nursing staff.”
  “Just pleasant people and that everyone knows what they are doing.”
3. Communication and kindness of doctors and nurses
  “Treat me as if you were to treat your parents.”
  “Good communication between staff, especially during shift change.”
4. Privacy and comfort
  “A single room -- quiet and privacy.”
  “I wouldn’t want to share a room. I think privacy is important when you’re a patient and I know on the floor you don’t get that option.”
5. Responsiveness of doctors and nurses
  “Getting seen quick and fixed quick.”
  “For the doctors to take care of what needs to be taken care of.”
6. System efficiency and coordination of care
  “To have everything done as quickly as possible so I can go home as quickly as possible.”
  “Get me back on my feet and get me home, get out of the way for other patients who need the spot.”

#### Patient Preferences

We asked patients to choose between the ICU and the floor if they were the patient in the scenario. Thirty-three patients (10.9%) chose the ICU, 133 (44.0%) chose the floor, and 136 (45.0%) had no preference.

The open-ended questionnaire provided additional information for each of these choices.

1. Prefer the ICU
  “ICU since you get better care.”
  “ICU… because there is a possibility of surgery. I would like to be watched very good.” “ICU… because a bleed in the brain is pretty serious and the brain is a very vital organ.”
2. Prefer the floor
  “The floor would be fine… If I just have a headache and there is nothing that seems to be critical at the time then I think the floor would be fine unless something changed.”
  “I guess I could go to the floor, my gut tells me that the floor would be faster. Faster in terms of getting in/out of the hospital.”
  “Just the floor, I don’t need the ICU because I know the difference between the floor and ICU, and I wouldn’t really qualify for the ICU and I don’t need one to one nursing care especially if it’s only for observation.”
  “For a small bleed, floor. The ICU is meant for people who need intensive care. A small bleed isn’t intensive.”
  “Probably the floor… if I wasn’t immediately dying I don’t see a reason to go to the ICU if it’s just to be observed and watched.”
  “Well I wouldn’t want to be in the ICU, because it doesn’t sound like I’m that sick, that’s space that could keep somebody else.”

#### Perceptions of Care

We asked specific questions on the closed-ended questionnaire aimed at evaluating the perceived differences in admission to the ICU and the floor (Table 3).

The open-ended questionnaire revealed three summative categories of differences between admission locations. This list outlines these categories with representative patient quotations.

1. ICU is more closely monitored/more responsiveness
  “The only thing I would guess is better more people available to you for more immediate responses in the ICU.”
  “The ICU they give you a lot more attention, they are watching you, it feels like constantly and they treat you a lot better in the ICU than they do on the floor at this hospital.”
2. There are more nurses and doctors on the ICU
  “More practitioners in the ICU than the floor.”
  “Your treatment is like one nurse for every two patients.”
3. No differences between the ICU and the floor
  “I don’t think there is very much of a difference… if you are hospitalized in the ICU, you’re in a bed just like you are on the floor and doctors come around and they see everybody in your unit or wherever you’re at and they spend time with you… it’s the same amount of time, you’re getting the same treatment as anybody one else, but in intensive care they do get a little more people who are watching them 24/7 but the doctors are not doing any more for them than they are doing for you on the floor.”

We asked patients to estimate the daily charge for the ICU and the floor. The median estimate for the ICU was $2000 (IQR $1000 to $5000) and the floor was $1000 (IQR $500 to $3000). When asked if they would be willing to pay more for an ICU, 76/298 (26%) responded “yes” and would pay a median of $500 (IQR $200 to $2000) more. Seventy-nine (26.9%) patients strongly agreed that doctors should consider bed availability when making admission decisions, while 37 (12.5%) of patients strongly agreed that doctors should consider cost (eTable 2).

## DISCUSSION

To our knowledge, this is the first study to evaluate patient perceptions and preferences regarding hospital admission location. We distributed closed- and open-ended questionnaires to ED patients to investigate patient preferences and perceptions of hospital care, specifically differences between ICU and hospital floor admissions. The questions referred to a hypothetical scenario of mild TBI and were based on hospitalization factors from the validated HCAHPS survey. The overall objective of the closed-ended questionnaire was to quantitatively evaluate any general trends in responses, while the open-ended questionnaire aimed to capture a more personal level of responses and identify any themes that may have been missed by the closed-ended questionnaire.

There were a number of interesting results from the survey. It was surprising that the majority of patients, given the hypothetical clinical scenario, preferred to be admitted to the floor (44%) or had no preference regarding admission location (45%), while only 11% preferred admission to the ICU. This was despite 51% of patients responding that admission to the ICU would result in overall better care (9% felt the admission to floor would result in better care and 40% responded no difference). These results challenge the notion that patients generally prefer more “intensive care” and suggest that hospitalization factors other than direct clinical care may influence patients’ overall preference on admission location.

Our results also suggest that patient-centered factors of hospitalization, such as physician and nurse availability and the ability for family and friends to visit, were consistently more important to patients than systemic/logistical factors of hospitalization, such as length and cost of hospitalization or length of time in the ED waiting for an in-patient bed. Future initiatives to improve patients’ hospitalization experiences should consider emphasizing improving patient-centered factors over logistical factors of hospitalization. Patient-centered initiatives, such as more liberal visitation hours 12 – 13 or including families during rounds 14 – 15, also may be easier to implement than improving systemic/logistical factors of hospitalization such as decreasing ED waiting time. 16

Regarding patients’ perceptions of hospitalization, patients thought admission to the ICU compared to admission to the floor, would result in more attention from doctors and nurses, have more privacy, be more expensive, be more difficult for family and friends to visit, and have longer waiting times for an in-patient bed in the ED. However, a substantial proportion of patients felt there was no difference between the ICU and the floor when asked about various factors of hospitalization. It is important to differentiate between patient perceptions of care and experiences of care. Prior studies have shown that perceptions of care may not be accurate of actual care 17 and may influence patient satisfaction more than actual experiences. 18 Future work may be directed towards evaluating the relationships between patient perceptions, experiences, and satisfaction of hospitalization. 19

Patients are admitted to the ICU for observation for a wide-range of clinical conditions despite being at low risk for requiring critical care interventions. 7 Prior studies demonstrated limited clinical benefit of ICU admission for low-risk patients with drug overdoses, 20 post-carotid endarterectomy, 21 angioedema, 22 gastrointestinal hemorrhage, 23 and traumatic intracranial hemorrhage. 8 – 9, 11 Given the limited clinical benefit in these low-risk patients, other factors such as patient preferences, cost, and resource availability should be considered. Appropriate utilization of ICU resources, which is costly (one-third of acute hospital charges) and limited (8% of hospital beds), 24 is important in the era of escalating healthcare costs. 25

Traditionally, admission decisions are unilateral – decisions are made by the clinician with minimal input from patients and/or their caregivers. However, while frequently not categorized as such, the decision to admit patients to the ICU or hospital floor is an intervention with risks and potential benefits to the patient and their caregivers. In addition, these decisions impact the healthcare system as a whole and indirectly impact other patients through the use of limited resources. Shared decision-making should be considered in situations where an intervention is not considered “standard” (defined as “virtual unanimity among patients about the overall desirability…of the outcomes”). 26 The role of shared decision-making in decisions regarding level of care during hospital admission is unclear. Patients and their caregivers may not comprehend the nuances between the ICU or the floor or they may not want to participate in the decision-making process. 27 Also, the addition of patient input may lead to disagreements with physicians and patients with unclear methods of resolution. However, as we move to a delivery-of-care model that is more patient-centered with increasing implementation of shared decision-making, a better understanding of patient perceptions and preferences of care will be of greater importance.

### Limitations

These results should be interpreted in the context of several limitations. Our results are based on a cross-sectional survey of ED patients. We sampled stable, low-acuity ED patients who could be conveniently queried in the ED waiting room from a single-center and thus their responses may not be generalizable to the ED population as a whole. In addition, participants who agreed to participate in the survey may be different from those who refused. Patient responses were based on a specific clinical scenario of a patient with mild TBI. Results may differ if the clinical scenario were different or if we surveyed patients currently experiencing the clinical scenario. Quotations were categorized into common themes; however, some quotations may be categorized into more than one theme. Sixty-eight percent and 30% of patients were previously admitted to the hospital and the ICU respectively. Thus, many subjects have limited prior personal experience with understanding the differences between the ICU and the floor.

### Conclusion

Based on a hypothetical scenario of mild TBI, the majority of patients preferred admission to the floor or had no preference compared to admission to the ICU. Humanistic factors such as availability of doctors and nurses and the ability to interact with family appear to have a greater priority than systematic factors of hospitalization.

## Figures and Tables

**Figure:**
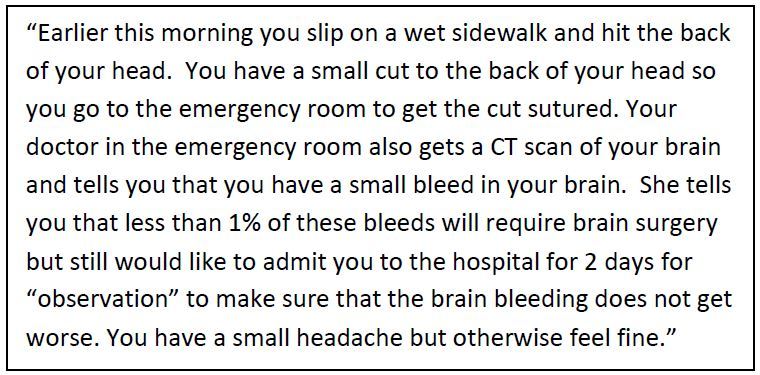
Hypothetical clinical scenario. *CT*, computed tomography

**Table 1:** Patient characteristics, n=332.

Characteristic	n, %
Age, mean (standard deviation)	44.3 (15.0)
Male	143/329, 43.5%
Race	
American Indian or Alaska Native	10/325, 3.1%
Asian	11/325, 3.4%
Black or African American	60/325, 18.5%
Native Hawaiian or Pacific Islander	8/325, 2.5%
White	175/325, 53.9%
Other	61/325, 18.8%
Hispanic/Latino	64/322, 19.9%
Education	
No high school	40/325, 12.3%
High school	72/325, 22.2%
Some college	101/325, 31.1%
2 year college degree	44/325, 13.5%
4 year college	47/325, 14.5%
Graduate degree	21/325, 6.5%
Health insurance	
No insurance	39/317, 12.3%
County insurance	14/317, 4.4%
Medi-Cal	57/317, 18.0%
Medicare	54/317, 17.0%
Health maintenance organization	70/317, 22.1%
Preferred provider organization	53/317, 16.7%
Other	25/317, 7.9%
Don’t know	5/317, 1.6%
Emergency severity index	
1 (Highest acuity: life or limb threatening)	0/311, 0%
2 (High risk situation)	97/311, 31.2%
3 (Multiple resources anticipated)	174/311, 56.0%
4 (One resource anticipated)	35/311, 11.3%
5 (No resources anticipated)	5/311, 1.6%
Seen on weekend	61/329, 18.5%
Seen at night (7 pm to 7 am)	57/326, 17.5%
Arrival mode	
Emergency medical services	16/329, 4.9%
Private car	194/329, 59.0%
Walk-in	110/329, 33.4%
Unknown	9/329, 2.7%
Prior emergency department visit	252/317, 79.5%
Prior hospital admission	210/317, 66.3%
Prior intensive care unit admission	91/315, 28.9%
Self-reported general health	
Excellent	12/326, 3.7%
Very good	84/326, 25.8%
Good	107/326, 32.8%
Fair	77/326, 23.6%
Poor	46/326, 14.1%

**Table 2:** Importance of hospitalization factors, n=302.

Factor	Responded as “very important” a, n (%)	Responded as “most important” b, n (%)
Family and friends can visit	251/302 (83.1)	42/296 (14.2)
Nurse availability	242/302 (80.1)	26/296 (8.8)
Physician availability	238/302 (78.8)	159/296 (53.7)
Privacy	220/302 (72.9)	8/296 (2.7)
Ability to sleep well at night	212/302 (70.2)	15/296 (5.1)
Length of time in the emergency department	207/302 (68.5)	18/296 (6.1)
Cost of hospitalization	187/302 (61.9)	22/296 (7.4)
Length of hospitalization	177/302 (58.8)	6/296 (2.0)

a see eTable 1 for complete breakdown of responses.

b six patients had missing responses.

**Table 3:** Perceptions of care, n=302.

Where do you think…	ICU n (%)	Floor n (%)	No difference n (%)
... you will receive overall better care?^a^	153 (51.0)	27 (9.0)	120 (40.0)
… your family and friends will have an easier time visiting you?^b^	21 (7.1)	191 (64.1)	86 (28.9)
... you will receive more attention and care from your doctors?^c^	201 (67.2)	20 (6.7)	76 (26.1)
... you will receive more attention and care from your nurses?^c^	174 (58.2)	32 (10.7)	93 (31.1)
... it costs more per day?^b^	253 (84.9)	6 (2.0)	39 (13.1)
... a bed will become available earlier from the ER?^d^	60 (20.2)	161 (54.2)	76 (25.6)
... you will stay longer in the hospital?^d^	110 (37.0)	107 (36.0)	80 (26.9)
... you will have more privacy?^c^	157 (52.5)	71 (23.8)	71 (23.8)
... you will get better sleep?^b^	103 (34.6)	72 (24.2)	123 (41.3)

*ICU,* intensive care unit; *ER*, emergency room

a two missing responses.

b four missing responses.

c three missing responses.

d five missing responses.
