# Supplementary material for: Patient Admission Preferences and Perceptions
Source: West J Emerg Med. 2015 Oct 20;16(5):707–14. doi: 10.5811/westjem.2015.7.27458 (PMC4644039; doi:10.5811/westjem.2015.7.27458)
Supplement: Supplementary file 1 [file wjem-16-707-s001.pdf]

Appendix. Closed Questionnaire

PATIENT INSTRUCTIONS
Please read the following scenario and answer the following questions.

SCENARIO
Earlier this morning you slip on a wet sidewalk and hit the back of your head. You have a small cut to the back of your head so you go to the emergency room to get the cut sutured. Your doctor in the emergency room also gets a CT scan of your brain and tells you that you have a small bleed in your brain. She tells you that less than 1% of these bleeds will require brain surgery but still would like to admit you to the hospital for 2 days for "observation" to make sure that the brain bleeding does not get worse. You have a small headache but otherwise feel fine.

QUESTIONS
Preferences (Questions 1 to 10)
For the following questions, please answer as if you were the patient in this scenario.

1. While you are in the hospital, how important is it to you that your family and friends can visit you?
   [ ] Very important
   [ ] Somewhat important
   [ ] A little important
   [ ] Not important at all

2. While you are in the hospital, how important is it to you that your doctor is available to see you frequently?
   [ ] Very important
   [ ] Somewhat important
   [ ] A little important
   [ ] Not important at all

3. While you are in the hospital, how important is it that to you that your nurse is available to see you frequently?
   [ ] Very important
   [ ] Somewhat important
   [ ] A little important
   [ ] Not important at all

4. While you are in the hospital, how important to you is the cost of your hospitalization?
   [ ] Very important
   [ ] Somewhat important
   [ ] A little important
   [ ] Not important at all

5. While you are in the hospital, how important is it that to you how quickly you leave the emergency room and get to your hospital room?
   [ ] Very important
   [ ] Somewhat important
   [ ] A little important
   [ ] Not important at all

6. While you are in the hospital, how important to you is how long you end up staying in the hospital?
[ ] Very important
[ ] Somewhat important
[ ] A little important
[ ] Not important at all

7. While you are in the hospital, how important to you is your privacy?
[ ] Very important
[ ] Somewhat important
[ ] A little important
[ ] Not important at all

8. While you are in the hospital, how important to you is your ability to sleep well at night?
[ ] Very important
[ ] Somewhat important
[ ] A little important
[ ] Not important at all

9. Assuming your health and safety would be the same, which of the following is most important factor during your hospitalization? (check only 1 option)
[ ] ability for my family and friends to visit
[ ] availability to doctors
[ ] availability to nurses
[ ] cost of the hospitalization
[ ] how quickly I will get a bed upstairs
[ ] privacy
[ ] ability to sleep at night
[ ] other: ________________

10. Assuming your health and safety would be the same, where would you prefer to be admitted to?
[ ] the intensive care unit (ICU)
[ ] a regular hospital bed (also called the ward or hospital floor)
[ ] no preference

Perception of care (Questions 11 to 24)

11. Where do you think you will receive overall better care?
[ ] ICU
[ ] Floor
[ ] No difference

12. Where do you think your family and friends will have an easier time visiting you?
[ ] ICU
[ ] Floor
[ ] No difference

13. Where do you think you will receive more attention and care from your *doctors*?

[ ] ICU
[ ] Floor
[ ] No difference

14. Where do you think you will receive more attention and care from your *nurses?*
[ ] ICU
[ ] Floor
[ ] No difference

15. Where do you think it costs more per day?
[ ] ICU
[ ] Floor
[ ] No difference

16. Where do you think a bed will become available earlier from the ER?
[ ] ICU
[ ] Floor
[ ] No difference

17. Where do you think you will stay longer in the hospital?
[ ] ICU
[ ] Floor
[ ] No difference

18. Where do you think you will have more privacy?
[ ] ICU
[ ] Floor
[ ] No difference

19. Where do you think you will get better sleep?
[ ] ICU
[ ] Floor
[ ] No difference

20. How much do you think the hospital will charge (write one value) for a night in the intensive care unit (ICU) (not including medications and surgeries)?
$______________

21. How much do you think the hospital will charge (write one value) for a night in a regular hospital bed (ward or hospital floor) (not including medications and surgeries)?
$______________

22. If an ICU bed charges more than a regular hospital bed, would you be willing to pay more per day to be in an ICU bed?
[ ] No
[ ] Yes
--------> If yes:
How much more would you be willing to pay per day to be admitted to an ICU bed (write one value)?
$______________

23. My doctor should think about how long I will have to wait for a bed before he or she decides where to admit me.

[ ] Strongly agree
[ ] Agree
[ ] Neutral
[ ] Disagree
[ ] Strongly disagree

24. My doctor should think about how much a hospital bed costs per night before he or she decides where to admit me.

[ ] Strongly agree
[ ] Agree
[ ] Neutral
[ ] Disagree
[ ] Strongly disagree

Background

25. How would you rate your general health?

[ ] Excellent
[ ] Very good
[ ] Good
[ ] Fair
[ ] Poor

26. Please indicate your race (mark one).

[ ] American Indian or Alaska Native
[ ] Asian
[ ] Black or African American
[ ] Native Hawaiian or other Pacific Islander
[ ] White
[ ] Other (Please specify: ____________________)

27. Are you Hispanic/Latino(a)?

[ ] Yes
[ ] No

28. What is the highest level of education you completed?

[ ] Did not finish high school
[ ] Graduated from high school or have GED
[ ] Some college
[ ] 2-year college degree (Associate’s)
[ ] 4-year college degree (Bachelor’s)
[ ] Graduate degree (Master's, PhD, MD, JD)

29. If you have health insurance, what type of insurance do you have?
[ ] I do not have insurance
[ ] County insurance (In Sacramento, it is called CMISP)
[ ] Medi-cal (Medicaid)
[ ] Medicare
[ ] HMO plan (Health Maintenance Organization)
[ ] PPO plan (Preferred Provider Organization)
[ ] Other (please specify: ____)
[ ] I don't know

30. Not counting today, have you previously been a patient in the emergency room?
[ ] No
[ ] Yes

31. Have you previously been admitted to the hospital floor (any hospital) overnight?
[ ] No
[ ] Yes

32. Have you previously been admitted to the intensive care unit (ICU) at any hospital overnight?
[ ] No
[ ] Yes

Appendix. Open questionnaire

QUESTIONS: Open Ended
Please ask the patient the following questions and take notes and record:

1. If you were the patient in the scenario, would you prefer to be in the ICU or the floor? Why?

2. How do you think hospitalization in the ICU versus on the floor differ?

3. If you had to be admitted to the hospital, what factors are important to you?
