# Supplementary material for: Patient Admission Preferences and Perceptions
Source: West J Emerg Med. 2015 Oct 20;16(5):707–14. doi: 10.5811/westjem.2015.7.27458 (PMC4644039; doi:10.5811/westjem.2015.7.27458)
Supplement: Supplementary file 2 [file wjem-16-707-s002.pdf]

eTable 1. Hospitalization factors, n=302				
**While you are in the hospital, how important…**	Very, n (%)	Somewhat, n (%)	A little, n (%)	Not at all, n (%)
... is it to you that your family and friends can visit you?	251 (83.1)	38 (12.6)	9 (3.0)	4 (1.3)
... is it to you that your doctor is available to see you frequently?	238 (78.8)	60 (19.9)	4 (1.3)	0 (0)
... is it that to you that your nurse is available to see you frequently?	242 (80.1)	55 (18.2)	5 (1.7)	0 (0)
... to you is the cost of your hospitalization?	187 (61.9)	74 (24.5)	14 (4.6)	27 (8.9)
... is it that to you how quickly you leave the emergency room and get to your hospital room?	207 (68.5)	78 (25.8)	12 (4.0)	5 (1.7)
... to you is how long you end up staying in the hospital?	177 (58.8)	76 (25.2)	34 (11.3)	14 (4.7)
... to you is your privacy?	220 (72.8)	62 (20.5)	20 (6.6)	0 (0)
... to you is your ability to sleep well at night?	212 (70.2)	78 (25.8)	11 (3.6)	1 (0.3)
