# Supplementary material for: Patient Admission Preferences and Perceptions
Source: West J Emerg Med. 2015 Oct 20;16(5):707–14. doi: 10.5811/westjem.2015.7.27458 (PMC4644039; doi:10.5811/westjem.2015.7.27458)
Supplement: Supplementary file 3 [file wjem-16-707-s003.pdf]

eTable 2. Perceptions of care, n (%); n=302	
**Q23: My doctor should think about how long I will have to wait for a bed before he or she decides where to admit me.**	
Strongly Agree	79/294 (26.9)
Agree	90/294 (30.6)
Neutral	62/294 (21.1)
Disagree	37/294 (12.6)
Strongly Disagree	26/294 (8.8)
**Q24: My doctor should think about how much a hospital bed costs per night before he or she decides where to admit me.**	
Strongly Agree	37/296 (12.5)
Agree	56/296 (18.9)
Neutral	72/296 (24.3)
Disagree	71/296 (24.0)
Strongly Disagree	60/296 (20.3)
